# Influence of the Ripening Stage and Extraction Conditions on the Phenolic Fingerprint of ‘Corbella’ Extra-Virgin Olive Oil

**DOI:** 10.3390/antiox10060877

**Published:** 2021-05-30

**Authors:** Anallely López-Yerena, Antonia Ninot, Núria Jiménez-Ruiz, Julián Lozano-Castellón, Maria Pérez, Elvira Escribano-Ferrer, Agustí Romero-Aroca, Rosa M. Lamuela-Raventós, Anna Vallverdú-Queralt

**Affiliations:** 1 Department of Nutrition, Food Science and Gastronomy, XIA, Faculty of Pharmacy and Food Sciences, Institute of Nutrition and Food Safety (INSA-UB), University of Barcelona, 08028 Barcelona, Spain; naye.yerena@gmail.com (A.L.-Y.); nuriajimenru@gmail.com (N.J.-R.); julian.lozano@ub.edu (J.L.-C.); mariaperez@ub.edu (M.P.); lamuela@ub.edu (R.M.L.-R.); 2 Institute of Agrifood Research and Technology (IRTA), Fruit Science Program, Olive Growing and Oil Technology Research Team, 43120 Constantí, Spain; Antonia.Ninot@irta.cat (A.N.); agusti.romero@irta.cat (A.R.-A.); 3 CIBER Physiopathology of Obesity and Nutrition (CIBEROBN), Institute of Health Carlos III, 28029 Madrid, Spain; eescribano@ub.edu; 4 Laboratory of Organic Chemistry, Faculty of Pharmacy and Food Sciences, University of Barcelona, 08028 Barcelona, Spain; 5 Pharmaceutical Nanotechnology Group I+D+I Associated Unit to CSIC, Biopharmaceutics and Pharmacokinetics Unit, Department of Pharmacy and Pharmaceutical Technology and Physical Chemistry, Institute of Nanoscience and Nanotechnology (IN2UB), Faculty of Pharmacy and Food Sciences, University of Barcelona, 08028 Barcelona, Spain

**Keywords:** polyphenols, malaxation, crushing size, oleocanthal, oleacein

## Abstract

The ancient ‘Corbella’ olive variety from the center-north of Catalonia is being recovered to obtain quality extra-virgin olive oil (EVOO) with unique organoleptic properties. The aim of this work was to determine the effect of agronomic and technical factors on the phenolic fingerprint of EVOO and to establish the optimum harvesting time and crushing and malaxation conditions for ‘Corbella’ olives. Therefore, three different ripening indices (0.3, 1.2, and 3.2) and three crushing temperatures (10, 18, and 25 OC) were studied. Additionally, a factorial design to optimize the phenolic concentration of the EVOO was developed, applying a range of sieve diameters (4 and 6 mm), and malaxation time (30 and 60 min) and temperature (27, 32, and 37 °C). The phenolic profile was analyzed by ultra-high performance liquid chromatography coupled to mass spectrometry in a tandem detector. The level of secoiridoids, the major phenolic compounds in the oil, was higher when using olives harvested earlier. Oleuropein aglycone and ligstroside aglycone were degraded during crushing at high temperatures, resulting in the formation of oleacein and oleocanthal. The best processing conditions in terms of total phenolic content were found to be 30 min of malaxation at 37 OC, the crushing size not having any affect.

## 1. Introduction

The demand for extra virgin olive oil (EVOO) is increasing due to growing consumer awareness of the health benefits of the Mediterranean diet, in which it is the main source of dietary fat [1]. The nutritional and health-promoting properties of EVOO [2,3] are mainly correlated with its highly bioactive components, such as monounsaturated fatty acids (55–83%), unsaponifiable compounds (1–2%), and soluble or hydrophilic compounds, including α-tocopherol, phenolic compounds, and other compounds with antioxidant properties [4]. In 2012, the European Food Safety Authority (EFSA) recognized that a daily consumption of 5 mg hydroxytyrosol and/or its derivatives (e.g., the oleuropein complex and tyrosol) per 20 g of olive oil afforded protection of LDL particles from oxidative damage and maintenance of a normal blood HDL-cholesterol concentration [5].

Since this recognition, the scientific community and olive oil industry have focused considerable efforts on optimizing the EVOO extraction process, searching for a balance between yield and quality [6]. To date, many studies have shown that the phenolic composition of EVOO depends on a highly complex multivariate interaction between agronomic, biochemical, and technological factors [7,8,9,10]. Firstly, the composition of the olives depends on their genetic origin, the geographical area of cultivation and its climate, and agronomic practices such as fertilization and water availability [11,12]. Then, the parameters associated with EVOO quality can be controlled by regulating the main variables of mechanical extraction (temperature, oxygen, enzymatic activities, and the disruption level of olive cell tissues) [13,14]. In this context, crushing is a determinant factor in the release and activation of endogenous enzymes (β-glycosidases and oxidoreductases), whereas malaxation is crucial for the modulation of the enzymatic activity [14,15,16,17] and production of high quality EVOO. During malaxation, the chemical composition of the oil undergoes considerable changes due to partition phenomena between oil and water, as well as reactions catalyzed by the fruit enzymes released during crushing [13].

Given the above, the main novelty of this work lies in the study of both agronomic and technological variables to obtain EVOO with a high content of phenolic compounds. For this purpose, the objectives of this study were (i) to determine the effect of three different ripening indices (RI, 0.3, 1.2, and 3.2) on the phenolic profile of EVOO, (ii) to evaluate changes in polyphenol content of EVOO obtained at three crushing temperatures (10, 18, and 25 °C), and (iii) to optimize the malaxation parameters (time and temperature) and crushing size to obtain an EVOO with higher phenolic compounds content. Regarding this last objective, a factorial design was developed to optimize the time (30 and 60 min) and temperature (27, 32, and 37 °C) of malaxation, applying two sieve diameters (4 and 6 mm). The study was carried out with olives from the ‘Corbella’ cultivar, native to Bages in the center-north of Catalonia, an ancient variety that was abandoned and at risk of extinction. EVOO produced exclusively with ‘Corbella’ olives has a powerful, fruity, and balanced taste. In recent years, centenary ‘Corbella’ olive trees have been recovered to promote the cultivation of this autochthonous variety and offer the market ecological products with added value.

## 2. Materials and Methods

### 2.1. Chemicals and Reagents

Regarding the secoiridoids, oleocanthal (≥95% purity) was purchased from Merck (Darmstadt, Germany), while oleacein and oleuropein aglycone (≥90 and 95% purity, respectivley) from Toronto Research Chemical Inc. (ON, Canada). Luteolin was acquired from Sigma-Aldrich (Madrid, Spain), ferulic acid and apigenin from Fluka (Buchs, Switzerland), and hydroxytyrosol from Extrasynthese (Genay, France). Hexane, methanol, and acetonitrile were purchased from Sigma-Aldrich. Ultrapure water was obtained using a Milli-Q purification system (Millipore, Bedford, MA, USA).

### 2.2. Plant Material 

Olive fruits were hand collected from mature olive trees of the ‘Corbella’ cultivar in the Oil Migjorn farm in Bages, Barcelona Province, Spain. The olives used to study the crushing temperature, crushing size, and malaxation were cultivated under controlled deficit irrigation conditions, whereas those used for the ripening study were obtained from non-irrigated trees. On the day of harvesting, the olives were sent to the IRTA laboratories in Constantí, Tarragona, where, after a health assessment, they were treated as described below.

### 2.3. Oil Extraction

All the fruit samples were processed using an Abencor analyser (Abengoa S.A., Seville, Spain), which reproduces the industrial process on a laboratory scale. The fruits were milled using a stainless-steel rotatory hammer mill operating at 3000 rpm and equipped with different sieves, depending on the experiment. The resulting olive paste was immediately passed into a malaxation unit with blades spinning at 50 rpm. The time and temperature of this step were set depending on each experiment. A basket centrifuge (3000 rpm for 3 min) was used to centrifuge the kneaded olive paste without addition of hot water or talcum. After centrifugation, the oil was decanted and stored in amber glass bottles at −20 °C in darkness and without headspace until analysis.

#### 2.3.1. Ripening Study

The fruits were collected at 3 different stages of maturity and, following the method described by Uceda and Frías [18], the 3 RI were calculated to be 0–1, 1–2, and 2–3. For each RI, the olives were crushed in a 5 mm diameter sieve, and the olive paste was placed in a 28 °C malaxator for 30 min without the addition of talcum powder or hot water. The centrifugation and storage conditions were as described above (Section 2.2).

#### 2.3.2. Crushing Temperature Study

Healthy olives were stored for two hours in a fridge (7 °C) and then washed using clean water heated to three different temperatures: (10, 18, and 25 °C) in a water bath. Once the desired temperature was reached, fruits were crushed in a 5 mm diameter sieve, and the resulting paste was placed in a malaxator at 18–19 °C for 30 min without adding either talcum or water. It was then centrifuged at 3.000 rpm for 3 min without adding water. The EVOO obtained was separated and stored under the conditions described above (Section 2.2).

#### 2.3.3. Crushing Size and Malaxation Study

The olive oil was extracted following a factorial model with three factors: crushing size (4 and 6 mm), malaxation temperature (27, 32, and 37 °C), and malaxation time (30 and 60 min). The trials were performed using a 3.3 kg batch of drupes. Three representative olive samples, each weighing a minimum of one kilogram, were processed, and the corresponding EVOOs were obtained using an Abencor analyser (Abengoa S.A., Seville, Spain). The olive fruits were milled using a stainless-steel hammer mill equipped with a 4- and 6-mm sieve that was operated at 3000 rpm. The resulting olive paste was immediately kneaded in a mixer at 50 rpm for 30 or 60 min at 27, 32, or 37 °C. The centrifugation and storage conditions were as described above (Section 2.2).

### 2.4. Phenolic Compound Composition

After a liquid–liquid extraction, the identification and quantification of phenolic compounds were carried out by liquid chromatography coupled to mass spectrometry in tandem mode (LC-MS/MS), as described in our previous studies [11,19]. Samples were analyzed using an Acquity ^T M^ UPLC (Waters; Milford, MA, EUA) system with an API 3000 triple-quadruple mass spectrometer (PE Sciex, Concord, Ontario, Canada). The system was equipped with a turbo ion spray source. Separation of phenolic compounds was conducted using an Acquity UPLC^®^ BEH C_18_ column (2.1 × 50 mm, i.d., 1.7 µm particle size) and Acquity UPLC^®^ BEH C_18_ Pre-Column (2.1 × 5 mm, i.d., 1.7 µm particle size) (Waters Corporation^®^, Wexford, Ireland).

The liquid–liquid extraction was carried out following procedures described previously [19]. For this purpose, 0.5 g of EVOO was dissolved in hexane (oil/hexane 1:2, *w*/*v*) in a 10 mL centrifuge tube and shaken for 1 min. For the phenolic compound extraction, 2 mL of MeOH: H_2_O (4:1 *v*/*v*) was added and the samples were homogenized for 1 min. The two phases were separated by centrifugation (3000 rpm, 4 °C, 4 min). The methanolic fraction was separated and subjected to a second cleaning using hexane, while the remaining phenolic compounds in the hexane fraction were extracted by a further addition of MeOH: H_2_O (4:1 *v*/*v*). The extracts were shaken and centrifuged under the same conditions as previously described. The two methanolic fractions were pooled and reduced to dryness with N_2_, redissolved in 800 μL of ACN, and stored at −80 °C prior to analysis.

The separation of oleocanthal, oleacein, ligstroside, and oleuropein aglycone was performed following the procedure proposed by Lozano-Castellon [19]. MeOH (A) and H_2_O (B) were used as mobile phases, both with 0.1% of formic acid. An increasing linear gradient (*v/v*) of B was used (t (min), %B), as follows: (0, 100); (2, 100); (4.75, 46.4); (4.9, 100); (5.9, 0); (6, 100); (6.5, 100), at 0.6 mL/min, 5 µL, and 50 °C of constant flow rate, injection volume, and column temperature, respectively. The separation of other polyphenols was achieved following procedures described previously [11] using H_2_O with 0.2% acetic acid (A) and ACN (B) as mobile phases. An increasing linear gradient (*v*/*v*) of B was used (t (min), %B), as follows: (0, 5); (2.5, 5); (12.5, 40); (12.6, 100); (13.5, 100); (13.6, 5); (15, 5), at 0.4 mL/min, 5 µL, and 40 °C of constant flow rate, injection volume, and column temperature, respectively.

The ionization was achieved using an electrospray (ESI) interface operating in negative ionization mode (M–H), and the compounds were monitored in the multiple monitoring mode (MRM). The system was controlled by Analyst version 1.4.2 software supplied by Applied Biosystems. The calibration curves were prepared in refined olive oil and were linear over the concentration range 0–20 mg/mL using hydroxytyrosol, apigenin, luteolin, oleocanthal, oleacein, and oleuropein aglycone as standards.

### 2.5. Statistical Analyses

The normality of the data was tested by the Q–Q plot diagnostic graphic. After viewing the results, all subsequent tests were non-parametric. In the ripening and crushing study, to assess differences between groups, a Kruskal–Wallis test was performed, and the post-hoc test was the pairwise Mann–Whitney U-test. To investigate the main effects of the investigated factors, as well as to determine possible interactions between them, phenolic concentration analysis data were subjected to three-way analysis of variance (ANOVA), with 2 crushing size, malaxation temperature, and malaxation duration as factors (3 malaxation temperatures × 2 malaxation durations × 2 crushing size × 3 replicates). Means were compared by least significant difference (LSD) test, at the level of *p* < 0.05. All the statistical analyses were performed with R Project for Statistical Computing 3.6.0. 

## 3. Results and Discussion

### 3.1. Ripening Study

**Figure 1:**
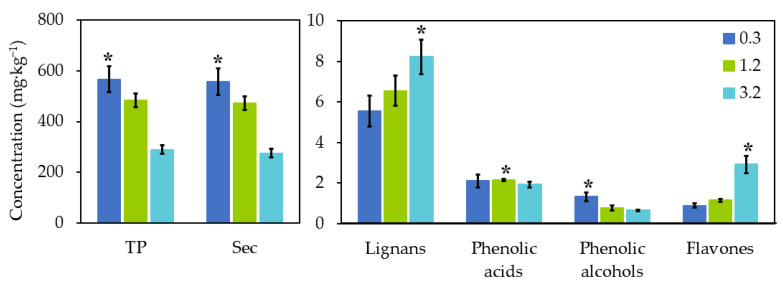
Effect of the ripening index on the total phenolic content and the phenolic composition of EVOO. TP: total phenols; SEC: secoiridoids. * indicates significant differences between phenolic concentration (*p* < 0.05).

The effects of ripening on the phenolic concentrations are shown in Figure 1. The total phenolic content was affected negatively by ripening stage, decreasing up to 49% at 3.2 of RI (from 566.74 ± 51.45 to 289.48 ± 17.43 mg·kg^−1^) (*p* ≤ 0.05). The same behaviour was observed for the secoiridoids’ and phenolic alcohols; concentration. In contrast, the maximum concentration of flavones and lignans was reached at 3.2 of RI, whereas the highest phenolic acid content was obtained with olives from the second harvest with an RI of 1.2 (2.16 ± 0.06 mg·kg^−1^). 

The RI of the olive fruits affected the total amount of phenolic compounds in the EVOO, which was 43.64% lower (*p* ≤ 0.05) when produced from the third harvest. This could be caused by endogenous enzymes in the olives, such as esterases, polyphenol oxidases, and β-glucosidases, which degrade phenols during the maturation process [13,14,16]. These results are in agreement with the literature [16,20,21,22], including a study carried out with ‘Frantoio’ and ‘Manzanilla’ EVOO, in which a gradual decrease in phenolic compounds was observed from the first to the fifth harvest [20]. In another study, however, this tendency did not begin below an RI of 2.5–3 [16]. It has also been suggested that the phenolic content is more influenced by the cultivar than the degree of fruit maturity [23]. It should be mentioned that the fruit of some olive cultivars ripens at an uneven rate, and therefore the same harvest will contain olives of variable ripeness [24], rendering the RI a poor descriptor for analysis. The ‘Corbella’ cultivar exhibits a very uniform ripening pattern [25].

The RI effect on the concentrations of individual phenolic compounds is shown in Table 1. The concentration of secoiridoids, the prevalent phenolic group found in EVOO and associated with many of its benefits [26], was greatly affected by the harvest time, as in other studies [13,27,28]. All the secoiridoids decreased as the RI increased, except for hydroxyoleuropein aglycone. The explanation may be that the enzymatic degradation of oleuropein and ligstroside results in a higher concentration of the aglycone. 

Among the phenolic alcohols, hydroxytyrosol decreased with maturation. In contrast, hydroxytyrosol acetate increased, probably because it can form part of more complex compounds such as oleacein, oleuropein, and verbascoside, and is released when they are broken down [29]. Due to its powerful radical scavenging activity, hydroxytyrosol acetate plays an important role in the anti-inflammatory effect of EVOO and is able to downregulate cyclooxygenase-2 expression [30]. In the case of hydroxytyrosol, which is also a degradation product, its reduced concentration could be caused by its higher lability. This effect is of interest, as according to EFSA, the consumption of at least 5 mg of hydroxytyrosol and its derivatives is required to protect LDL particles from oxidative damage [5]. Therefore, to preserve the health benefits of EVOO, a higher RI is not recommended, as the resulting reduction of hydroxytyrosol far exceeds the increase in the acetate derivative.

In the case of flavones, an upward trend was observed, as in other studies [31,32]. Bengana et al., 2013 [33] reported an increase in the flavonoid concentration in EVOO produced from olives in the first period of maturation, although levels began to decline when the harvest was in December. This tendency was not observed in our work, as all the olives were collected before the end of October. The glycosidic forms of flavones found in the drupelet become more soluble in oil during ripening, when the bond with the sugar is broken down by β-glucosidase. Thus, with flavones in oil being retained to a higher degree than in water during the extraction, oils from late season olives have a higher flavone content. However, other studies report that while luteolin increased, apigenin levels remained unchanged in ‘Hojiblanca’, ‘Picual’, and ‘Picudo’ oil varieties [34] or even decreased in the ‘Arbequina’ variety [35]. Such variable maturation behaviour can be attributed to differences in composition and endogenous enzymes among varieties [23,36]. Additionally, accurate comparisons of results from different studies are not always possible, as some refer only to the harvest date but not the RI [23].

The level of phenolic acids showed a slight decrease from the first to the second RI, as reported elsewhere [32]. In contrast, other studies have found an increase, associated with the activity of hydrolytic enzymes in the drupelet [28,37]. 

Among the lignans, pinoresinol increased by approximately 40.75% over the three ripening stages, possibly because of the degradation of the coexisting acetoxypinoresinol [38], whose levels were below the detection limit. Pinoresinol is a very stable compound and is preserved during ripening. Lignans are reported to have an inhibitory activity against cancer cell growth and antiestrogenic effects [39,40].

### 3.2. Crushing Temperature Study

**Figure 2:**
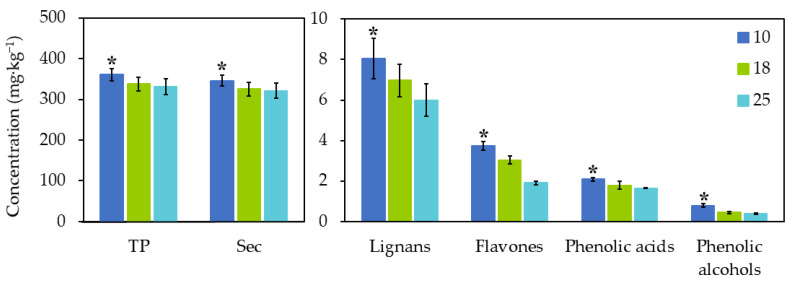
Effect of crushing temperature on the total phenolic content and the phenolic composition of EVOO. TP: total phenols; Sec: secoiridoids. * indicates significant differences between phenolic concentration (*p* < 0.05).

The crushing of the olive fruit is the first operation in the extraction of olive oil. The disruption of fruit tissues releases the oil and triggers the enzymatic reactions that shape the profile and concentration of phenolic and volatile compounds [41]. Table 2 presents the effect of the three different crushing temperatures on the phenolic profile of EVOO. As shown in Figure 2, the total phenols decreased with higher temperatures, with a significant difference observed between 10 and 18 °C (360.70 ± 15.00 to 337.55 ± 17.00 mg·kg^−1^) (*p* ≤ 0.05), but not between 18 and 25 °C. All the phenolic groups (secoiridoids, phenolic alcohols, flavones, phenolic acids, and lignans) followed the same trend (*p* ≤ 0.05). Similarly, a study on olive oil of the ‘Cima di Bitonto’ cultivar, applying three crushing temperatures (12, 16, and 20 °C), reported a gradual decrease in the phenolic content [42]. The overall quality of EVOO, attributed to its content of minor compounds, can therefore be modulated by using the appropriate crushing conditions [43]. A study carried out by Caponio and Catalano [44] showed that higher temperatures in the crusher during olive processing led to a shorter preservation of the oils, mainly attributed to a higher degree of auto-oxidation.

The secoiridoids oleuropein aglycone and ligstroside aglycone underwent enzymatic degradation at higher crushing temperatures, resulting in the formation of oleacein and oleocanthal. During crushing and malaxation, β-glucosidases and esterases are released from the olive flesh and pit and can readily interact with phenols, the reaction kinetics increasing with the temperature [45]. A high concentration of oleocanthal and oleacein in EVOO might be desirable due to their health properties [8,26].

The levels of the phenolic alcohols hydroxytyrosol and hydroxytyrosol acetate decreased by 50.77 and 49.02% at 18 and 25 °C, respectively. Hydroxytyrosol underwent a great change between 10 and 18 °C, whereas the acetate derivative declined progressively. The EVOO concentration of flavones at 25 °C was 48.93% lower than at 10 °C. Similarly, in a study of ‘Ayvalik’ oils, malaxation at 27 °C produced oils with a higher concentration of luteolin than at 37 and 47 °C [46]. Accordingly, higher processing temperatures reduce the content of flavones in olive oil.

The effect on the phenolic acids was comparable to that observed in total phenols, with the most substantial degradation recorded at 18 °C, and only a slight further reduction at 25 °C. These results reflect that at lower temperatures phenolic acids are stable or degrade very little. Finally, although less drastically, lignans also declined with higher temperatures, in contrast with another study that found their concentration was unaltered by changes in temperature during malaxation [47].

### 3.3. Crushing Size and Malaxation Study

Considerable research has been carried out to clarify which factors most affect the concentration of phenolic compounds in EVOO, with the aim of maximizing its health properties. In this context, many studies have investigated the impact of malaxation temperature and time, together with either the time of harvest [48], extraction techniques [49], or the degree of ripeness [27,50], on the concentration of total and individual phenols. Crushing size has been evaluated to a lesser extent, often together with the hammer mill rotor speed [41,51]. Therefore, by analyzing the influence of the temperature and time of malaxation, as well as the crushing size, our study opens a new three-factor approach to optimizing the phenol content of EVOO.

The effect of malaxation temperature (27, 32, and 37 °C), malaxation time (30 and 60 min) and crushing size (4 and 6 mm) on the total and individual phenol content is presented in Table 3. In general, the malaxation temperature affected the content of total and individual phenols, except for ligstroside aglycone and luteolin (*p* > 0.05), whereas the duration was the only factor that modified the concentration of all the compounds analyzed and consequently the total polyphenol concentration (*p* < 0.05). Crushing size did not affect the concentration of ligstroside and oleuropein aglycone, luteolin, and pinoresinol (*p* > 0.05).

Concerning the factorial interaction effects, temperature*time modified the concentration of hydroxyelenolic acid, oleocanthal, hydroxytyrosol, and apigenin, but to a low extent (*p* < 0.05). Mixing temperature * crushing size affected the final concentration of up to 70% of phenolic compounds and the total polyphenols. Finally, malaxation time* crushing size significantly affected the concentration of total polyphenols, hydroxyelenolic acid, oleocanthal, oleacein, oleuropein aglycone, and hydroxytyrosol (*p* < 0.05).

**Figure 3:**
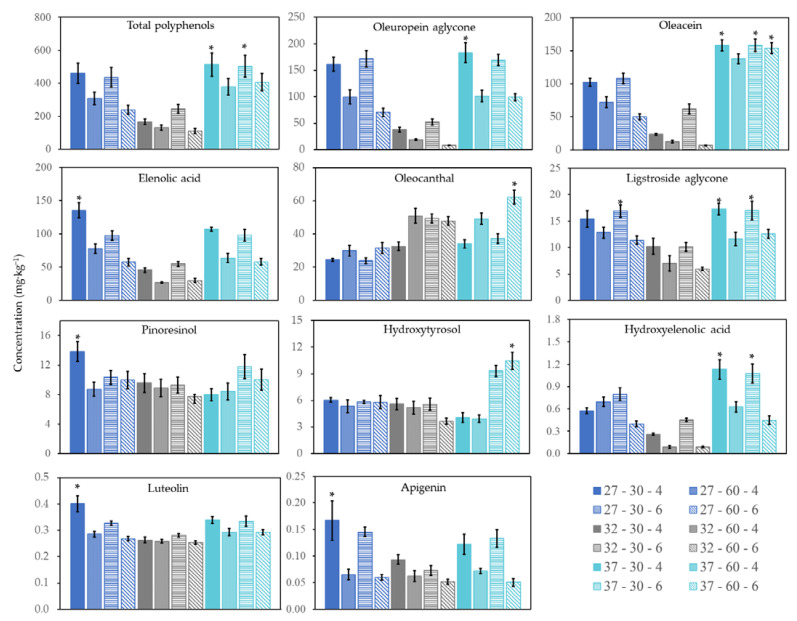
Effect of malaxation temperature (27, 32, and 37 °C), malaxation time (30 and 60 min), and crushing size (4 and 6 mm) on the total phenol content and the phenolic profile of EVOO. * indicates significant differences between phenolic concentration (*p* < 0.05).

The temperature*time*size interaction affected the content of total phenols and oleocanthal, oleacein, oleuropein aglycone, hydroxytyrosol, and apigenin. The factorial design showed that the EVOOs with the highest phenolic contents were obtained after 30 min of malaxation at 37 °C, the amount being 513.43 ± 70.89 mg·kg^−1^ for a crushing size of 4 mm and 503.21 ± 66.72 mg·kg^−1^ for 6 mm; conversely, the lowest content was obtained at 32 °C (Figure 3). The final concentration of phenolic compounds in EVOO probably depends on the equilibrium between their transfer to the oil phase and oxidative degradation [52]. A similar positive influence of a higher malaxation temperature has been reported [17,49,52], although some studies found that temperatures beyond 30 °C resulted in a lower phenolic concentration due to an enhanced activity of endogenous peroxidase (POD) and polyphenol oxidase (PPO) enzymes [17,47,53,54,55,56]. In addition, a short malaxation time increased the total phenol content (Figure 3), as in other studies [49,56,57], whereas a long malaxation time is rarely reported to have a positive effect [58]. The benefits of a brief malaxation can be attributed to a reduced activity of the hydrolytic and oxidative enzymes (POD and PPO) and less diffusion of the phenols to the water phase [56,57]. In our study, it was also demonstrated that the crushing size (4 and 6 mm) barely affected the total phenol content when the malaxation conditions were 37 °C for 30 min, in accordance with results in the literature [41,43,50].

Regarding the secoiridoids, the highest concentration of oleuropein aglycone was obtained after 30 min of malaxation at 37 °C applying a crushing size of 4 mm (183.27 ± 18.99 mg·kg^−1^). On the other hand, three treatments provided the best conditions to obtain an EVOO rich in ligstroside aglycone and oleacein. In the case of ligstroside aglycone, two treatments with the highest temperature (37 °C, 30 min, and 4 or 6 mm) and one with the lowest (27 °C, 30 min, and 6 mm) gave the best results. These findings are in line with the analysis of the factorial and interaction effects, where only the malaxation time significantly modified the concentration of this aglycone (*p* < 0.05). In the case of oleacein, its EVOO concentration was favored by the highest temperature (37 °C, 30 or 60 min, and 4 or 6 mm) (Figure 3). The optimal conditions for oleocanthal were 60 min at 37 °C with a crushing size of 6 mm. Finally, the highest concentration of elenolic acid (135.52 ± 11.33 mg·kg^−1^) was obtained using conditions of 27 °C, 30 min, and 4 mm and hydroxyelenolic acid with 37 °C, 30 min, and 4 or 6 mm.

Other studies have reported that higher malaxation temperatures may (i) increase the partition coefficient between oil and water phases in olive paste [59], (ii) reduce the activity of PPO enzymes [17], (iii) boost the release of phenols from the cell wall of polysaccharides and other olive tissues [15,17], and (iv) increase enzymatic activity of β-glucosidases and esterases. β-glucosidases catalyze the hydrolysis of oleuropein and ligstroside, leading to their aglycone forms, and also contribute to the formation of oleacein and oleocanthal in combination with esterases [60]. The observed increase in concentration of most of the secoiridoids can be attributed to the reasons mentioned above.

Regarding duration, a longer malaxation noticeably reduced the content of secoiridoids, except for oleocanthal. An excessive malaxation time can result in undesired reactions catalyzed by PPO and phenoloxidases [17,57,61] by increasing olive paste exposure to air. Moreover, prolonged contact between the paste and water favors the diffusion of phenols into the aqueous phase [57,61]. Previous studies have reported a similar increase in oleocanthal, with other secoiridoids showing either a weak dependence on the malaxation duration [27,50] or a decreasing trend over time [48,57]. In summary, the oils with the highest secoiridoid content, except for elenolic acid, were produced by a malaxation temperature of 37 °C. These results are in agreement with Boselli et al. (2009), but again they disagree with the conclusions of other studies that describe a negative impact of higher temperatures on secoiridoids, probably due to an enhanced rate of degradation [52].

The hydroxytyrosol content was also affected by extraction conditions (Figure 3), the highest concentration (10.45 ± 0.98 mg·kg^−1^) being obtained by malaxation at 37 °C for 60 min and a crushing size of 6 mm. Contradictory results have been reported in the literature, in that a longer olive paste malaxation reduced the hydroxytyrosol concentration [57], as did a higher malaxation temperature [27].

Flavones (apigenin and luteolin) and lignans (pinoresinol) followed a similar pattern, concentrations being lowest at 32 °C and highest at 27 °C, in both cases with a malaxation time of 30 min and crushing size of 4 mm. Similar results have been recently reported for flavones [31,52,62], whereas other authors describe that the duration of malaxation has little effect on flavone and lignan content [57]. Other studies have found opposite trends, reporting that the pinoresinol content increased with temperature and malaxation time [31].

## 4. Conclusions

The total phenol content in the EVOO extracted from ‘Corbella’ olives decreased with fruit maturity, but flavones, hydroxyoleuropein aglycone, hydroxytyrosol acetate, and lignans increased. A negative correlation was found between the total phenols and higher crushing temperatures, although individual phenolic compounds such as oleocanthal and oleacein increased. In summary, to obtain a ‘Corbella’ EVOO rich in phenolic compounds, an early harvest and low crushing temperature should be employed. On the contrary, if a higher concentration of specific phenols such as oleocanthal or oleacein is desired, due to their health properties, a moderate crushing temperature should be used.

Regarding malaxation conditions, the results suggest that a higher temperature (37 °C) increased the total phenol content and the concentration of the most relevant phenols. A short malaxation time (30 min) enhanced the total phenols and oleuropein and ligstroside aglycone, whereas a longer process (60 min) increased oleocanthal concentration. Finally, a clear trend was not observed with respect to crushing size.

Based on these results, future research could be focused on studying the correlation between the enzymatic activity and ripening index or how the malaxation temperature affects olives collected at different times.

## Figures and Tables

**Table 1:** Phenolic compound contents (mg·kg^−1^) of EVOO produced from ‘Corbella’ olives with different crushing temperatures.

Group/Compound	Ripening Index		
	0.3 ± 0.07	1.2 ± 0.17	3.2 ± 0.27
Total Polyphenols	566.74 ± 51.45 ^a^	483.72 ± 26.65 ^b^	289.48 ± 17.43 ^c^
Secoiridoids	556.81 ± 51.45 ^a^	473.07 ± 26.17 ^b^	275.72 ± 17.43 ^c^
Elenolic acid	272.12 ± 9.71 ^a^	228.82 ± 16.30 ^b^	125.52 ± 9.89 ^c^
Hydroxyelenolic acid	8.68 ± 0.38 ^a^	8.79 ± 0.29 ^a^	4.32 ± 0.21 ^b^
Hydroxyoleuropein aglycone	1.11 ± 0.09 ^b^	1.16 ± 0.07 ^b^	2.03 ± 0.07 ^a^
Ligstroside aglycone	15.07 ± 0.84 ^a^	11.92 ± 0.47 ^b^	9.00 ± 0.51 ^c^
Methyloleuropein aglycone	0.92 ± 0.04 ^a^	0.88 ± 0.02 ^a^	0.87 ± 0.02 ^a^
Oleacein	94.25 ± 6.29 ^a^	78.72 ± 4.48 ^b^	34.06 ± 2.06 ^c^
Oleocanthal	9.14 ± 1.20 ^a^	6.84 ± 0.31 ^b^	2.36 ± 0.21 ^c^
Oleuropein aglycone	155.51 ± 5.71 ^a^	135.95 ± 2.98 ^a^	97.56 ± 3.75 ^b^
Phenolic alcohols	1.34 ± 0.21 ^a^	0.78 ± 0.11 ^b^	0.66 ± 0.04 ^c^
Hydroxytyrosol	1.27 ± 0.08 ^a^	0.68 ± 0.06 ^b^	0.46 ± 0.05 ^c^
Hydroxytyrosol acetate	0.08 ± 0.02 ^b^	0.11 ± 0.02 ^b^	0.21 ± 0.02 ^a^
Flavones	0.90 ± 0.09 ^c^	1.15 ± 0.08 ^b^	2.92 ± 0.41 ^a^
Apigenin	0.49 ± 0.01 ^c^	0.82 ± 0.06 ^b^	2.23 ± 0.2 ^a^
Luteolin	0.41 ± 0.02 ^b^	0.32 ± 0.04 ^b^	0.69 ± 0.04 ^a^
Phenolic acids	2.12 ± 0.32 ^ab^	2.16 ± 0.06 ^a^	1.93 ± 0.15 ^b^
*m*-coumaric acid	1.53 ± 0.04 ^a^	1.47 ± 0.03 ^a^	1.42 ± 0.03 ^b^
*p*-coumaric acid	0.59 ± 0.05 ^ab^	0.69 ± 0.04 ^a^	0.50 ± 0.04 ^b^
Lignans	5.57 ± 0.76 ^b^	6.56 ± 0.75 ^b^	8.24 ± 0.85 ^a^
Pinoresinol	5.57 ± 0.76 ^b^	6.56 ± 0.75 ^b^	8.24 ± 0.85 ^a^

Different letters within the same row mean significant differences (*p*-value < 0.05) according to the Kruskal–Wallis test.

**Table 2:** Phenolic compound contents (mg·kg^−1^) of EVOO produced from ‘Corbella’ olives using different crushing temperatures.

Group/Compound	Crushing Temperature (°C)		
	10	18	25
Total Polyphenols	360.70 ± 15.00 ^a^	337.55 ± 17.00 ^b^	331.23 ± 20.00 ^b^
Secoiridoids	346.03 ± 14.00 ^a^	325.30 ± 17.00 ^b^	321.28 ± 19.00 ^b^
Elenolic acid	191.00 ± 8.00 ^a^	175.00 ± 10.00 ^ab^	171.00 ± 4.00 ^b^
Hydroxyelenolic acid	7.44 ± 0.50 ^a^	6.06 ± 0.50 ^b^	6.13 ± 0.30 ^b^
Hydroxyoleuropein aglycone	2.14 ± 0.08 ^a^	1.72 ± 0.10 ^b^	1.74 ± 0.08 ^b^
Ligstroside aglycone	10.00 ± 0.20 ^a^	8.96 ± 0.50 ^b^	8.47 ± 0.10 ^c^
Methyloleuropein aglycone	0.87 ± 0.01 ^a^	0.87 ± 0.01 ^a^	0.87 ± 0.00 ^a^
Oleacein	16.50 ± 0.60 ^c^	29.70 ± 2.00 ^b^	35.20 ± 1.00 ^a^
Oleocanthal	0.95 ± 0.09 ^c^	2.05 ± 0.1 ^b^	2.42 ± 0.20 ^a^
Oleuropein aglycone	117.10 ± 2.96 ^a^	97.20 ± 5.00 ^b^	95.10 ± 2.00 ^b^
Phenolic alcohols	0.79 ± 0.10 ^a^	0.45 ± 0.05 ^b^	0.39 ± 0.04 ^b^
Hydroxytyrosol	0.59 ± 0.04 ^a^	0.28 ± 0.03 ^b^	0.29 ± 0.04 ^b^
Hydroxytyrosol acetate	0.20 ± 0.02 ^a^	0.16 ± 0.01 ^b^	0.10 ± 0.01 ^c^
Flavones	3.74 ± 0.20 ^a^	3.04 ± 0.20 ^b^	1.91 ± 0.09 ^c^
Apigenin	2.85 ± 0.20 ^a^	2.28 ± 0.10 ^b^	1.43 ± 0.07 ^c^
Luteolin	0.89 ± 0.09 ^a^	0.67 ± 0.04 ^b^	0.49 ± 0.05 ^c^
Phenolic acids	2.08 ± 0.09 ^a^	1.79 ± 0.20 ^b^	1.65 ± 0.01 ^b^
*m*-coumaric acid	1.49 ± 0.04 ^a^	1.43 ± 0.04 ^ab^	1.38 ± 0.03 ^b^
*p*-coumaric acid	0.60 ± 0.06 ^a^	0.33 ± 0.02 ^b^	0.27 ± 0.04 ^b^
Lignans	8.06 ± 1.00 ^a^	6.97 ± 0.80 ^ab^	5.99 ± 0.80 ^b^
Pinoresinol	8.06 ± 1.00 ^a^	6.97 ± 0.80 ^ab^	5.99 ± 0.80 ^b^

Different letters within the same row mean significant differences (*p*-value < 0.05) according to the Kruskal–Wallis test.

**Table 3:** Effect of malaxation temperature, malaxation time, crushing size, and their interaction on phenolic compound contents (mg·kg^−1^) in EVOO produced from ‘Corbella’ olives.

	MTE		MT		CS		MTE × MT		MTE × CS		MT × CS		MTE × MT × CS	
Compound	*p* Value	F Value	*p* Value	F Value	*p* Value	F Value	*p* Value	F Value	*p* Value	F Value	*p* Value	F Value	*p* Value	F Value
Total Polyphenols	<0.001	218.2	<0.001	96.7	0.0013	11.39	0.1351	2.29	<0.001	21.93	<0.001	32.83	<0.001	32.67
Elenolic acid	<0.001	34.4	<0.001	76.14	0.0179	5.92	0.1762	1.87	<0.001	37.95	0.2157	1.57	0.1909	1.75
Hydroxyelenolic acid	<0.001	103.58	<0.001	89.65	0.0019	10.59	0.0005	13.46	0.0445	4.21	0.0013	11.36	0.3988	0.72
Ligstroside aglycone	0.1559	2.07	<0.001	57.75	0.2206	1.53	0.4396	0.61	0.6632	0.19	0.3395	0.93	0.113	2.59
Oleacein	<0.001	1271.08	<0.001	147.99	<0.001	35.66	0.4081	0.69	0.0001	18.05	<0.001	66.25	<0.001	60.79
Oleocanthal	<0.001	432.54	<0.001	45.1	<0.001	30.78	0.0008	12.38	0.0001	18.95	<0.001	64.67	<0.001	71.49
Oleuropein aglycone	0.0002	15.88	<0.001	50.93	0.6893	0.16	0.6681	0.19	0.8331	0.04	0.0075	7.65	0.0053	8.39
Hydroxytyrosol	<0.001	46.77	<0.001	21.08	0.0032	9.44	0.023	5.44	<0.001	266.07	0.0036	9.16	0.0002	15.34
Apigenin	0.0007	12.92	0.0001	18.33	0.0191	5.8	0.0036	9.16	0.3479	0.89	0.2888	1.15	0.0103	7.02
Luteolin	0.1896	1.76	0.0171	6.02	0.3164	1.02	0.6117	0.26	<0001	25.01	0.1389	4.69	0.1310	2.34
Pinoresinol	0.0012	11.49	0.0223	5.5	0.1407	2.23	0.9473	<0.001	<0.001	32.07	0.3674	0.82	0.323	0.99

MTE: malaxation temperature; MT: malaxation time and CS: crushing size.

## Data Availability

Data is contained within the article.
